# Acute Kidney Injury Caused by Superior Mesenteric Artery Syndrome

**DOI:** 10.1155/2020/8364176

**Published:** 2020-04-09

**Authors:** Nur Ezzaty Mohammad Kazmin, Lydia Kamaruzaman, Zhiqin Wong, Voon Ken Fong, Rozita Mohd, Ruslinda Mustafar

**Affiliations:** ^1^Medical Department, National University of Malaysia Medical Centre, Kuala Lumpur, Malaysia; ^2^Nephrology Unit, Medical Department, National University of Malaysia Medical Centre, Kuala Lumpur, Malaysia; ^3^Gastroenterology Unit, Medical Department, National University of Malaysia Medical Centre, Kuala Lumpur, Malaysia

## Abstract

**Background:**

Superior mesenteric artery (SMA) syndrome is a rare cause of upper gastrointestinal obstruction leading to acute kidney injury (AKI).

**Methods:**

We report a case of 23-year-old army personnel who presented with persistent vomiting leading to severe hypokalaemia, metabolic alkalosis, and acute kidney injury resulting in cardiorespiratory arrest.

**Results:**

After successful resuscitation, he was supported with haemodialysis and aggressive electrolytes correction. He was repeatedly not able to tolerate nasogastric (NG) tube feeding and computerised tomography of abdomen was performed, and the diagnosis of SMA syndrome was made. Gastroscopy examination revealed duodenal ulcer at D1, pinhole D1-D2 junction, but there was no evidence of intraluminal mass or lesions leading to upper gastrointestinal obstruction. A nasojejunal tube was inserted to bypass the narrow segment of the duodenum, and he was put on nutritional support. He was subsequently weaned off dialysis support as his renal function gradually improved and later on normalised. He remains symptoms free, and he gained five kilograms in four months after discharge.

**Conclusions:**

SMA syndrome is a rare cause of upper gastrointestinal obstruction but should be considered as a differential diagnosis in a patient who presented with recurrent vomiting and AKI with metabolic alkalosis.

## 1. Introduction

Superior mesenteric artery (SMA) syndrome is one of the rare causes of upper gastrointestinal (GI) obstruction with reported a very low prevalence of 0.013–0.3% [1, 2]. It was first described following autopsy findings by Carl Freiherr von Rokitansky in 1842. SMA syndrome is characterised by extraluminal vascular compression of the third segment of the duodenum (D) between SMA and aorta, leading to complete or partial duodenal obstruction. Patients could present with symptoms varying from chronic abdominal discomfort lasting for months to recurrent vomiting and acute upper intestinal ileus [3]. Diagnosis is usually challenging and requires a high index of suspicion due to the nonspecific presentations and its rarity, while radiological imaging with computerised tomography (CT) is considered a gold standard for diagnosis. To date, limited cases had described the association of metabolic alkalosis in a patient with significant renal impairment [4–6]. Management includes supportive measures by nutrition supplementation to help weight gain and build retroperitoneal fat tissue, although the majority of patients will require surgical intervention to relieve the obstruction [7].

## 2. Case Report

A 23-year-old man who was previously fit army personnel presented to our emergency department (ED) with a history of intermittent postprandial vomiting and leg cramps for one month which had worsened two days before his presentation where he vomited for ten times a day. He also had no loin pain or haematuria. Clinically, he was alert, not pale, neither jaundice nor cachexic, and without any abnormality on physical examination. On arrival, his blood pressure (BP) was 130/73 mmHg, pulse rate of 92 beats per minute, and oxygen saturation of 98% under room air.

The initial blood investigations in ED showed severe metabolic alkalosis with hypokalaemia, hypocalcaemia, and renal impairment. Blood urea was 9.1 mmol/L, serum creatinine of 218.3 *μ*mol/L (eGFR 35.5 ml/min/1.73 m^2^), potassium of 1.6 mmol/L, and sodium of 126 mmol/L. Venous blood gas showed metabolic alkalosis with pH of 7.56, bicarbonate of 62 mmol/L, pCO_2_ of 73.3, mm Hg, pO_2_ of 29.1, and SO_2_ of 52.2%. Corrected serum calcium was 2.04 mmol/L while serum phosphate was 1.0 mmol/L. Full blood count showed haemoglobin of 12.2 g/L, total white blood cell count of 10.2 × 10^9^/L, and platelet of 302 × 10^9^/L. Meanwhile, C-reactive protein was 0.28 mg/dL. There were no baseline investigations for comparison and also no arterial blood gas results.

Shortly after reviewing him, he developed generalised tonic seizure followed by cardiac arrest with pulseless electrical activity. The blood pressure was not recordable; hence, cardiopulmonary resuscitation was carried out for a total of 70 minutes, with 14 direct current shocks delivered for ventricular tachycardia and fibrillation before he regained full and sustained recirculation. He was intubated and required inotropic support for persistent hypotension despite adequate volume resuscitation. He had 300 ml of urine following urinary bladder catheterisation after the resuscitation, which was most likely reflective of residual urine prior to the collapsed, but later he became anuric and was initiated on renal replacement therapy.

**Figure 1:**
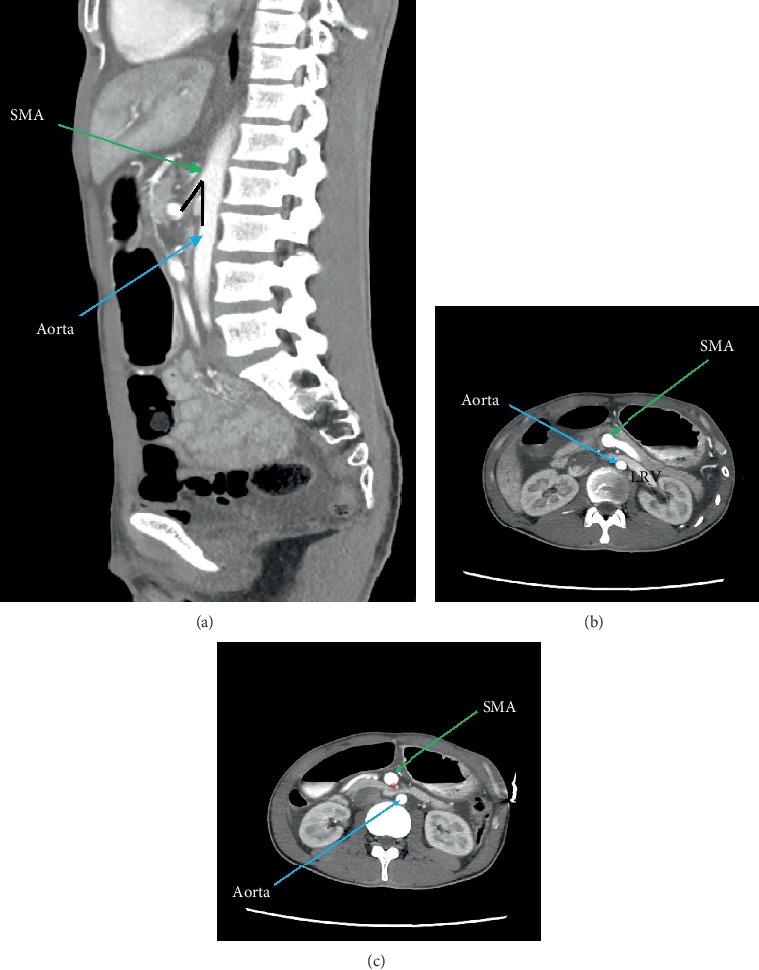
Contrasted CT abdomen image in (a) showing the narrow-angle created by the SMA anteriorly and descending aorta posteriorly. (b) and (c) showing dilated left renal vein (LRV) and third D3 (marked by^*∗*^), respectively, which are compressed by descending aorta posteriorly and SMA anteriorly.

Placement of nasogastric tube (NG) postintubation drained almost 2 L gastric content, and even after emptying, he was unable to tolerate feeding via the tube. He required multiple corrections for hypokalaemia and hypocalcaemia with intravenous potassium chloride and calcium gluconate infusion, respectively. CT thorax, abdomen, and pelvis was done and showed distended elongated stomach with air contrast level (Figure 1). There was no enhancing lesion or compression at the pylorus and antrum of the stomach. However, the D3 segment of the duodenum was significantly smaller in calibre at the precaval and preaortic region with lack of mesenteric fat. There was a reduced aortomesenteric angle measuring 21 degrees (normal: 25–60 degree) with reduced distance measuring 8 mm (normal: 10–28 mm). The contrast was seen opacifying the duodenum, jejunum and ileal loops.

He required intermittent haemodialysis and extubated after four days and could give more detailed histories which help to exclude the possibility of congenital related disorders or drug abuse which were not present. He was well before but gave a history of progressive weight loss with intermittent vomiting, which goes back to almost a year. Although it was not initially alarming, for the past two months, he lost 2 kilograms, despite normal appetite and no associated diarrhoea or vomiting. His body mass index on admission was 17.8 kg/m^2^ only with a weight of 48 kg and height of 1.64 m.

**Figure 2:**
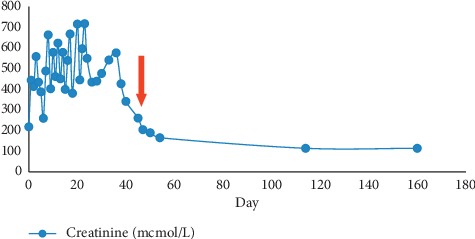
Showed serum creatinine trend over time, with a red arrow indicating the point when the patient was taken off haemodialysis support.

He had a full neurological recovery but remained intolerance to NG feeding. Gastroscopy examination showed Forrest 3 ulcer at D1, pinhole D1-D2 junction, which was dilated during the scope, and the nasojejunal (NJ) tube was inserted to facilitate oral feeding. He was given parenteral nutritional support throughout the hospital stay together with oral erythromycin ethylsuccinate as a prokinetic agent until he tolerates the tube feeding very well. He was then successfully discharged without the feeding tube after one month of admission. His renal function was also gradually recovered (Figure 2). He was taken off dialysis support after three months. With proper dietary advice, he gained five kilograms in a span of four months.

## 3. Discussion

SMA syndrome, apart from being very rare, can present with a broad spectrum of clinical manifestations and cause confusion with other causes of intestinal obstruction or motility disorders. The syndrome mostly affects female between the age 10 and 39 years [3] and those with low BMI < 18 kg/m^2^ or weight percentile for the height of <5% [8].

Its underlying pathophysiology involves narrowing of the aortomesenteric angle to 6 to 8 mm or reduction of its distance to 16 to 22 mm which results in extraluminal compression of the third part of the duodenum between the aorta and SMA. The predisposing factors can be divided into congenital or acquired. Some patients were born with shortened ligaments of Treitz causing the duodenum to be suspended nearer to the origin or SMA, thus prone for compression. More commonly, this condition is acquired as a result of retroperitoneal and mesenteric fat tissue loss in patients with chronic illness, dietary disorders, or severe malnutrition. Local pathology such as tumours or anatomical changes following injury, intra-abdominal surgical repair of aortic aneurysm, or spinal instrumentation has also been reported as predisposing factors [3].

So far, this condition has been described in patients with rapid weight loss with comorbidities or following surgery [9], but uncommon in a healthy individual. In this case, the patient did not have typical risk factors for the disease as he was previously fit army personnel with no known medical illness. Apart from the loss of retroperitoneal fat tissue due to progressive weight loss for past one year that probably attributed to his vigorous physical training, thus resultant to intermittent vomiting, CT scan, and gastroscopy examination with biopsy ruled out any local cause such as tumours that could contribute to the narrowing of the aortomesenteric angle.

Due to low suspicion index and delay in diagnosis of SMA syndrome, most of the patients present late with a long-standing history of upper abdominal discomfort, intermittent vomiting, and weight loss which could last up to 8 to 28 months [3, 9, 10]. Rarely, as in this case, the patient could present acutely with gastric dilation and upper intestinal ileus [3, 11–13] mimicking more common causes of acute vomiting such as gastroenteritis, pancreatitis, peptic ulcer disease, cholecystitis, mesenteric ischemia, and medication-related side effects [14, 15]. Mortality has also been reported due to gastric necrosis and perforation as a complication of the condition [11, 13].

AKI is a common presentation to medical attention and can be categorised by prerenal, renal, and postrenal causes. In general, most of the causes of AKI were apparent from histories such as volume loss following bleeding, burns, severe gastrointestinal (GI) loss, hypotension, sepsis, use of nephrotoxic drugs, or obstructive uropathy. Intrinsic renal causes of AKI such as glomerulonephritis and infiltrative disease are more difficult to diagnose and need a renal biopsy for confirmation.

On the other hand, AKI as a primary presenting feature of SMA syndrome remains uncommon, and only a few cases had reported AKI in association with the condition [12, 16, 17]. In severe GI obstruction with a massively distended stomach, AKI could occur as a result of increased abdominal compartment pressure, resulting in decreased renal artery perfusion as well as impaired venous drainage. Clinically, the patient presents with massively distended abdomen accompanied by tenderness and rigidity, as well as metabolic acidosis due to bowel hypoperfusion and ischemia [16]. Rarely, a patient can present with AKI due to simultaneous renal vein compression by superior mesenteric artery [8], a condition called nutcracker syndrome [18].

Nutcracker syndrome is also another possibility; however, apart from the incidental finding of left renal vein compression on the CT imaging which would suggest this phenomenon, the patient did not complain of any loin pain or haematuria prior to the presentation. Unfortunately, there was no urine sample sent on admission to check for the presence of any microscopic haematuria or sediment of red blood cells. Due to these limitations, the diagnosis of SMA syndrome is very clear based on the clinical findings; however, coexistence of nutcracker syndrome remains speculative in this case.

Recurrent vomiting in this case could lead to volume depletion and prerenal AKI, as well as metabolic alkalosis due to loss of hydrogen ion from the gastric juice. Apart from the initial prerenal insult due to hypovolemia from excessive vomiting and third space loss, this patient developed worsening AKI and subsequent oligoanuria likely due to hypotensive acute tubular necrosis (ATN) secondary to prolonged cardiorespiratory collapse and hypotension requiring renal replacement therapy.

Nevertheless, it was unusual for severe metabolic alkalosis to occur in a patient with significant renal impairment, as the latter is commonly associated with metabolic acidosis. Despite only two days history of excessive vomiting, this patient presented with life-threatening metabolic alkalosis causing neuromuscular irritability, seizure, and cardiac arrest likely due to a combination of low serum calcium, hypokalaemia, and hypoventilation induced cerebral hypoxemia. Raised venous pCO_2_ concentration on arrival suggests that somewhere following arrival and onset of the seizure, hypoxemia likely had occurred due to secondary hypoventilation in response to severe metabolic alkalosis [4].

Mechanism of severe metabolic alkalosis, in this case, is postulated to be initiated by hypergastrinemia following cholinergic stimulation due to acute gastric distention. This results in an increased volume of gastric juice produced, as well as hydrogen ion secreted, which was subsequently lost [4]. In a healthy patient, bicarbonate ion (HCO_3_) generated from hydrogen ion (H+) secreted from the parietal cell will be buffered by pancreatic HCO_3_ loss into the small intestine. However, in this case, there was a net HCO_3_ gained as gastric acid produced in the stomach failed to enter the small intestine to stimulate pancreatic secretion of HCO_3_. This HCO_3_ gain is maintained as the kidney is unable to excrete the excess HCO_3_ due to impaired glomerular filtration. At the same time, intravascular volume depletion secondary to excessive vomiting and third space loss causes secondary hyperaldosteronism which increases HCO_3_ reabsorption, causing worsening of metabolic alkalosis as well as renal potassium K+ ion loss [6]. Some cases had used low bicarbonate dialysate for the treatment of severe metabolic alkalosis [19]. However, here, haemodialysis using normal dialysate had successfully decreased serum bicarbonate concentration and brought down blood pH to normal.

In patients considered at risk for SMA syndrome, a high index of suspicion is needed to minimised delay in the diagnosis. Contrasted CT has been considered the gold-standard imaging to visualise aortomesenteric angle and distance accurately for diagnosis of SMA syndrome. CT will also be able to pick up local pathologies such as neoplasm or aneurysm that could be the cause of anatomical change and compression. On the other hand, upper endoscopic examination is usually required to exclude intraluminal causes of obstruction, including ulcers which could mimic the syndrome or develop as a complication secondary to reflux of gastric acids [3]. Rarely, an experienced endoscopist will be able to demonstrate pulsatile extrinsic compression which supports the diagnosis during the examination [2].

Management of SMA syndrome varies from medical to surgical procedures aiming to promote weight gain and to increase retroperitoneal fat tissue. Medical management via enteral nutrition given through a feeding tube bypassing the obstruction or a parenteral nutrition with or without prokinetic agents are the preferred strategies and has been proven to be successful in certain cases [20, 21]. Knee-chest or side-lying positional change after a meal had been tried to provide partial obstruction relief by widening the aortomesenteric angle [22, 23]. Surgical management, on the other hand, is usually reserved for patients not responding to medical management, and these include procedures such as gastrojejunostomy, open or laparoscopic duodeno-jejunostomy, and Strong procedure [8, 24].

The severities of complications reported are mainly related to delay in diagnosis, and these include electrolyte imbalances, dehydration, peptic ulcer, stomach perforation, aspiration pneumonia, pancreatitis, and even death [3, 24]. A patient whose diagnosis was picked up early during illness without significant complication usually has a good prognosis. In this case, although the patient was fortunate to be revived following cardiac arrest, he developed AKI and was temporarily dependent on haemodialysis support for seven weeks.

## 4. Conclusion

SMA syndrome is rare but should be considered as a differential diagnosis when a patient presents with recurrent vomiting and AKI with metabolic alkalosis. CT contrast is the gold-standard imaging for diagnosis together with an endoscopic examination to identify potential secondary causes for the narrowing of aortomesenteric angle causing D3 compression. Conservative medical management with nutritional support should be attempted in most patients, whereas surgical procedures are reserved for those not responding or came with complications that warrant immediate surgery. Generally, the prognosis of SMA syndrome, in this case, is good given that the condition is identified and intervenes early.
